# New Insight into Intestinal Mast Cells Revealed by Single-Cell RNA Sequencing

**DOI:** 10.3390/ijms25115594

**Published:** 2024-05-21

**Authors:** Erisa Putro, Alessia Carnevale, Caterina Marangio, Valerio Fulci, Rossella Paolini, Rosa Molfetta

**Affiliations:** Department of Molecular Medicine, Laboratory Affiliated to Istituto Pasteur Italia-Fondazione Cenci Bolognetti, Sapienza University of Rome, 00161 Rome, Italy; erisa.putro@uniroma1.it (E.P.); a.carnevale@uniroma1.it (A.C.); caterina.marangio@uniroma1.it (C.M.); valerio.fulci@uniroma1.it (V.F.); rosa.molfetta@uniroma1.it (R.M.)

**Keywords:** intestinal mast cells, gut inflammation, colorectal cancer

## Abstract

Mast cells (MCs) are tissue-resident immune cells distributed in all tissues and strategically located close to blood and lymphatic vessels and nerves. Thanks to the expression of a wide array of receptors, MCs act as tissue sentinels, able to detect the presence of bacteria and parasites and to respond to different environmental stimuli. MCs originate from bone marrow (BM) progenitors that enter the circulation and mature in peripheral organs under the influence of microenvironment factors, thus differentiating into heterogeneous tissue-specific subsets. Even though MC activation has been traditionally linked to IgE-mediated allergic reactions, a role for these cells in other pathological conditions including tumor progression has recently emerged. However, several aspects of MC biology remain to be clarified. The advent of single-cell RNA sequencing platforms has provided the opportunity to understand MCs’ origin and differentiation as well as their phenotype and functions within different tissues, including the gut. This review recapitulates how single-cell transcriptomic studies provided insight into MC development as well as into the functional role of intestinal MC subsets in health and disease.

## 1. Introduction

MCs arise from BM progenitors that enter the circulation and mature in peripheral tissues under the influence of microenvironment factors [1,2,3].

Mature MCs are tissue-resident innate immune cells that are present in all organs, particularly in skin, lung, and intestinal mucosa, and are distributed close to blood and lymphatic vessels and nerves. Thanks to their strategical localization and to the expression of a wide array of receptors, mature MCs act as tissue sentinels, able to firstly detect the presence of bacteria and parasites and to respond to different microenvironmental stimuli [4,5,6,7].

Their functions are mediated by the secretion of a vast array of biologically active molecules, including histamines and proteases that are stored in secretory granules and immediately released upon activation [8,9]. A plethora of newly synthesized lipid inflammatory mediators are secreted within hours [9]. Moreover, by releasing various cytokines and chemokines, MCs orchestrate the recruitment and activation of immune cells to the site of infection and regulate innate and adaptive immunity [10]. 

Among the main surface receptors, mature MCs are characterized by the expression of c-Kit (CD117) that upon interaction with its ligand (stem cell factor, SCF) regulates MC migration and activation [11], and the high-affinity receptor for immunoglobulin E (FcεRI) that orchestrates the IgE-mediated allergic reactions [12,13]. Indeed, cross-linking of FcεRI-bound IgE by multivalent antigens results in the release of granule-stored mediators such as histamine, accompanied by the generation of newly synthetized soluble mediators [13,14] and high quantities of extracellular vesicles, emerging as important players in intercellular communication [15,16]. 

Similarly, activation by anaphylatoxins or neuropeptides, including substance P, results in the degranulation of preformed mediators and the de-novo synthesis of chemokines/cytokines [17,18].

However, MCs also express a wide range of receptors that are pivotal in the host’s defense against pathogens, such as Toll-like receptors [19]. More recently, a selective expression of human mas-related G protein-coupled receptor X2 (MRGPRX2) and its mouse homologue, Mrgprb2, have been also reported [20]. This receptor can promote IgE-independent pseudo-allergic reactions by binding an array of host and microbial peptides, often generated from proteolytic cleavage of inactive precursors solely in inflamed tissues [20]. Thus, MC activation has been linked not only to allergy but also to other inflammatory conditions within different tissues, including the gut, where a cross-talk between MCs and nerves can also provide a neuroimmune network necessary to control local responses [21,22].

Notably, the presence of MCs has also been reported in several solid cancers accompanied by MC’s ability to shape the tumor microenvironment [23,24]. However, MCs can both orchestrate antitumoral responses, promoting the recruitment of other immune cells, and tumor progression favoring angiogenesis, lymphoangiogenesis, fibrosis, and metastasis [23,24,25]. 

**Figure 1:**
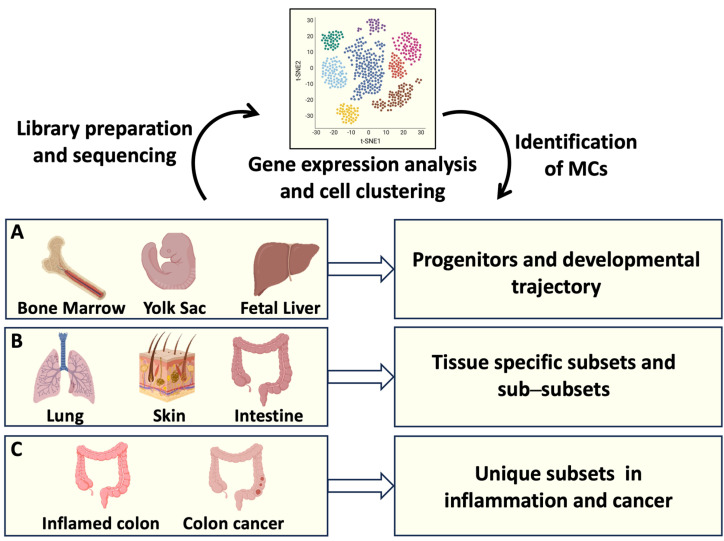
MC origin and tissue heterogeneity analyzed by single-cell RNA sequencing (RNAseq). Single-cell RNAseq offers the possibility to identify the transcriptomic profiles of several cells from a tissue of interest. Transcripts associated with individual cells are sequenced and analyzed, resulting in cell clustering based on gene expression. (**A**) Identification of MC progenitors in bone marrow, fetal liver, and yolk sac has defined MC developmental trajectories; (**B**) gene expression profiles of MCs resident in different organs have clarified MC tissue heterogeneity; (**C**) transcript analysis of intestinal MCs has provided information on MC phenotypical and functional plasticity in health and disease. Created using BioRender.com.

More recently, several aspects of MC biology have been solved thanks to the development of single-cell transcriptomic profiling technologies [26,27,28], as depicted in Figure 1. This novel approach was able to differentiate MCs from other immune cells, including basophils and eosinophils, and to reveal a unique mouse and human MC identity [26,27,28]. Moreover, the presence of distinct MC subsets in different connective tissues has been elucidated [29,30], revealing a high degree of MC heterogeneity [31,32]. 

However, MC’s phenotype and functions between and within different organs remain to be clarified. Moreover, how MC plasticity is shaped in different physiological and pathological conditions is largely unexplored. 

This review recapitulates data obtained from recent single-cell-based studies mainly focusing on intestinal MC subsets and their roles in health and disease. 

## 2. Transcriptomic Analysis and MC Development

A first transcriptomic study on MC differentiation by Saito and coauthors has been performed using progenitors derived from human umbilical cord blood and adult peripheral blood and has revealed a series of MC-specific genes, including *TPSAB1/2* (tryptase α1 and β1), *HDC* (L-histidine decarboxylase), and *CPA3* (carboxypeptidase A) [33]. 

More recently, a single-cell transcriptomic analysis revealed a temporal association between the appearance of FcɛRI and an MC signature in hematopoietic progenitor isolated from human peripheral blood [34].

Regarding the existence of a common progenitor between basophils and MCs, a first study integrating flow cytometric and transcriptomic data has been performed on primary BM-derived hematopoietic stem cells showing the presence of a cluster of cells expressing a set of common signature genes between basophils, eosinophils, and MCs [35]. Similarly, a single-cell RNA sequencing of progenitors from human cord blood identified an intermediate-stage progenitor that co-expresses gene modules of basophil, eosinophil, and MC lineages [36]. 

Notably, erythro-myeloid progenitors were found also in yolk sac, suggesting that, as happens in mice, human MCs arise from multiple compartments during and after embryogenesis [37]. 

More recently, by analyzing a single-cell dataset of human BM, Hamey and coauthors have provided a road map of MC and basophil development supporting the existence of a common progenitor until a bifurcation into the two specific cell lines [38]. However, a transcriptome analysis of mature human skin MCs demonstrated a unique MC transcriptional landscape, delineating a limited relation between MCs and basophils [39]. 

Thus, although human basophils and MCs express common marker genes (e.g., *HDC* and *FcεR*I), further studies are needed to explore in depth the transcriptional differences between them in order to better discriminate their developmental trajectories. 

In regard to similarities between distinct lineages, recent human and murine studies have suggested the existence of a hematopoietic progenitor with MC–erythrocyte potential [36,37,40,41,42]. However, the contributions of these progenitors to the resolution of infection-induced inflammation remain only poorly defined [42], as further discussed in Section 4.

## 3. Insights into Intestinal MC Origin and Phenotype through Single-Cell RNAseq

A tissue compartment in which MCs are particularly abundant is the gut. Intestinal MCs are involved in the maintenance of tissue homeostasis and at the same time act as sentinels of the host’s defense against different pathogens, orchestrating inflammation [22,43]. 

In the mouse, the small intestine represents a large reservoir of MC-committed progenitors (MCps) that are recruited by a mechanism involving α4β7 integrin and the CXC chemokine receptor-2 (CXCR2) [44]. As in all organs, critical signals for homing and maturation of MCps are also provided by SCF binding to c-Kit [3], and murine models with spontaneous mutations in white spotting locus coding for c-Kit have been used to identify and understand the contribution of MCs in several biological processes [45].

The study performed by Hamey and coauthors [38] offers valuable insights into the intricate process of MC differentiation in the gut, shedding light on the nuances of gene regulation during maturation. Focusing on peritoneal MCs, they observed that MC differentiation/maturation is characterized by the downregulation of β7 integrin, as well as the protease genes *Mcpt8* and *Gzmb* (*Granzyme B*). Notably, they also reported the upregulation of MC-specific protease genes including *Cpa3*, *Cma1*, *Mcpt4*, *Tpsb2*, *and Tpsab1*, revealing that their induction occurs in distinct temporal stages (*Cpa3* first, followed by *Tpsb2*, and finally *Tpsab1*) [38].

Mature MCs in the intestine are heterogeneous and comprise two main subsets that differ in localization and protease content [46]. In rodents, MCs are divided into mucosal MCs (MMCs) present in the intestinal lamina propria close to the epithelium and positive for Mctp1 and Mcpt2 proteases, and connective-tissue MCs (CTMCs) that reside in gut submucosa and are characterized by the expression of proteases Mcpt4-7 and Cpa3 as well as a higher amount of histamine and heparin compared to MMCs [47]. 

In humans, mucosal MCs present in lamina propria contain only tryptase in their granules (MC_T_), while MCs that predominate in the intestinal submucosa contain tryptase, chymase, Cpa3, and cathepsin G (MC_TC_) [48,49]. MCs that exclusively express chymase have also been identified as a rare population that resides in both lamina propria and submucosa [50]. Similarly, an intraepithelial MMC subpopulation has also been described in mice [51]. However, the role of these rare MC populations is still unclear. 

Recent advancements in RNAseq profiling technologies and fate mapping revealed different developmental origins between the two main MC populations in mice: MMCs originate from fetal hematopoietic stem cells and depend on adult stem cells for their replacement, while CTMCs originate from yolk sac and can self-maintain independently from BM-derived stem cells [41]. A similar conclusion came from the study by Gentek and coauthors revealing that CTMCs are maintained independently of adult hematopoietic stem cells [52].

In the human gut, a transcriptomic profile obtained by single-cell RNAseq analysis revealed that MCs express specific transcripts such as Vascular Endothelial Growth Factor A (*VEGFA*), the cytoskeleton component utrophin (*UTRN*), the chemokine receptor *CXCR4*, the aryl hydrocarbon receptor (*AHR*), and the interleukin 1 receptor-associated kinase 3 (*IRAK3*) [53]. However, this signature is not a unique characteristic of human intestinal MCs but is shared by MCs resident in bladders, lymph nodes, skeletal muscle, trachea, and tongue [53].

Furthermore, by integrating datasets from Mouse Cell Atlas derived from different tissues, the same authors demonstrated that CTMCs and MMCs are characterized by diverse gene signatures across organs [53]. In the gastrointestinal tract, MMCs, in addition to mucosal *Mcpt1* and *Mcpt2* protease genes, are characterized by a high expression of genes encoding adhesion molecules (*Itgae*, *Itga2a*, *Ly6e*) and the chemokine receptor *Cxcr1*, whereas CTMCs are enriched in *Cma1*, *Mcpt4*, *Tpsb2*, and *Cpa3* protease genes, *Ccl2* chemokine genes, and lipid metabolism genes (*Apoe*) together with the expression of *Mgbrb2* genes [53]. This latter result is in line with previous studies showing that the mouse ortholog of human MRGPRX is exclusively expressed on connective tissue-like MCs [26,54]. 

The origin of the two subsets was further explored, comparing mice at different ages [53]. CTMCs positive for Mrgprb2 were found in both neonatal pups and adults, while Mrgprb2^−^ Mcpt1^+^ MMCs were exclusively detected in adult mice, suggesting that Mrgprb2^+^ CTMCs originate embryonically, whereas Mrgprb2^−^ MMCs originate after birth. Moreover, the use of BM chimeras confirmed that the Mrgprb2^−^ MMCs are continuously renewed from BM progenitors, whereas the Mrgprb2^+^ CTMC population appears to be independent of BM-derived cells for turnover not only in the gut but also in the skin and peritoneal cavity [53]. Notably, CTMCs in distinct organs showed a high degree of differential gene expression [26], definitively demonstrating a microenvironment-dependent MC differentiation and suggesting that tissue-specific MC subsets exist beyond the traditional MMC/CTMC classification.

## 4. Deciphering Intestinal MC Function in Homeostasis and Inflammatory Conditions 

Intestinal MCs contribute to homeostasis by controlling physiological processes such as mucosal integrity and epithelial barrier activity [43,55]. Indeed, mice deficient in MCs or Mcpt4 protease have reduced small intestinal permeability and altered epithelial cell migration as well as intestinal morphology and tight junctions [55]. 

**Figure 2:**
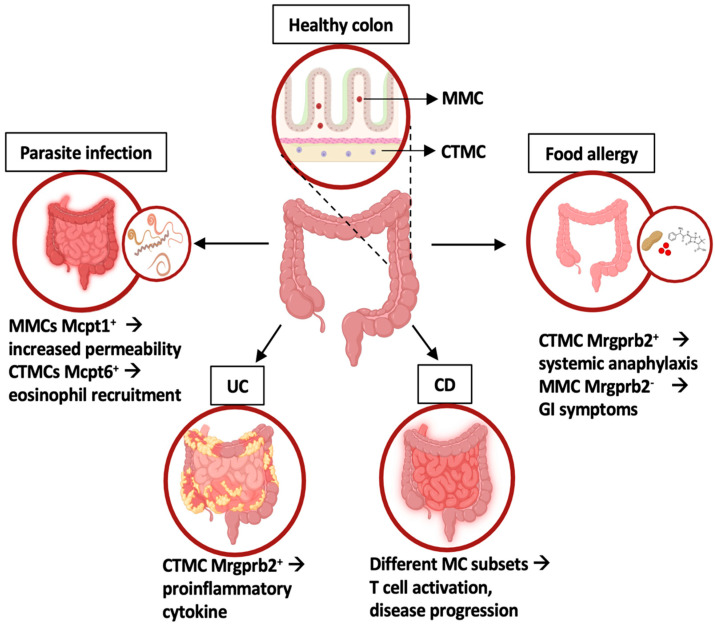
Intestinal MC phenotype and functions in homeostasis and inflammatory conditions. In healthy colons, two MC subsets have been identified: mucosal MC (MMC) and connective tissue-like MC (CTMC). In disease states, distinct MC subsets with unique gene expression profiles contribute to intestinal inflammation, as highlighted by different transcriptomic approaches. UC: ulcerative colitis; CD: celiac disease. Created using BioRender.com.

The crucial role of MCs in epithelial integrity is confirmed by their involvement in intestinal inflammatory conditions but also in food allergy and nematode infections (Figure 2).

During parasite infections, including *Trichinella spiralis* and *Trichuris muris*, MMCs are the main subset that increases in number due to a shift from a connective tissue-like phenotype to a mucosal phenotype characterized by the expression of the proteases Mcpt1 and Mcpt2 [56,57,58]. 

In particular, Mcpt1 appeared to be responsible for the degradation of occludin, thus increasing intestinal permeability and facilitating worm expulsion [56,57,58]. On the other hand, the connective tissue MC-specific tryptase Mcpt6 was shown to be required for eosinophil recruitment and the eradication of *T. spiralis* [59].

Notably, by single-cell RNAseq, Inclan-Rico and coauthors demonstrated that infection by *T. spiralis* induces the recruitment into the intestine of a hematopoietic progenitor with dual MC–erythrocyte potential [42], likely contributing to eradicate the infection and to alleviate blood loss. 

In addition to parasite infections, intestinal MCs are involved in IgE-mediated responses to food antigens contributing to both local and systemic development of food allergies [60,61]. 

An increase in MC number, mainly due to the expansion of intestinal MMCs, has been demonstrated both in humans and mice sensitized by food allergens, and correlates with the severity of symptoms [60,61]. However, using two common models of IgE-mediated food allergy, Benedé and Berin demonstrated that systemic anaphylaxis was uniquely associated with the activation of connective tissue-like MCs, while gastrointestinal manifestations of food allergy were associated with an increase of Mcpt1-expressing MCs together with a clear activation of both mucosal and connective tissue-like MCs [62]. More recently, Tauber and coauthors confirmed these findings, demonstrating that depletion of the Mrgprb2^+^ CTMC subset protects murine models from anaphylactic shock, while Mrgprb2^−^ MMCs in the gut are not implicated in anaphylaxis, despite being the first population to encounter the allergen [53]. 

MCs’ ability to rapidly sense and adapt to specific triggers including neuropeptides can explain the activated MC phenotype described in different human gastrointestinal disorders such as celiac disease, irritable bowel syndrome (IBS), and inflammatory bowel disease (IBD) [22,63]. IBDs are complex multifactorial diseases of the gastrointestinal tract, including ulcerative colitis (UC), triggered by environmental factors in genetically susceptible individuals [22]. Current therapies based on the use of monoclonal antibodies directed against cytokines offer amelioration and a prolonged period of remission but have important limitations. Indeed, more than 30% of patients do not initially respond to therapy, while others lose response over time [64,65]. Thus, new treatment strategies are needed.

Several studies have reported MCs’ accumulation in patients affected by celiac disease (CD) and UC, but their contribution in disease progression remained unclear. 

In this context, Atlasy and coauthors compared transcriptomic profiles of immune infiltrate isolated from small intestines of patients affected by active CD [66]. They found enrichment of different MC subsets in healthy and affected intestine: MCs that were more abundant in control patients showed a profile associated with “humoral immune response” and “positive regulation of B cell activation” biological processes, whereas MC clusters accumulated in active disease displayed a transcriptomic profile associated with “protein to ER process”, “antigen processing and presentation”, and “positive regulation of T cell-mediated cytotoxicity” processes [66], suggesting their active role in disease progression.

Similarly, Smillie and coauthors focused on colonic tissues from UC patients and healthy donors and, using single-cell RNAseq, mapped different cell circuits [67]. They identified 51 cell subsets (including epithelial, stromal, and immune cells) and revealed an increase in inflammatory-associated genes in UC patients compared with healthy volunteers. Notably, together with cytotoxic and regulatory T cells, a selective MC subset expressing the activation marker *CD69* was increased in inflamed tissues [67]. However, this MC subset was not further characterized in terms of protease content.

More recently, Chen and coauthors compared acutely inflamed and uninflamed UC tissue to establish the requirement of MRGPRX2-mediated MC activation in inflamed colonic tissues [68]. Using both bulk RNAseq and single-cell RNAseq, they reported a key role for adrenomedullin (*ADM*) and its proteolytic product, PAMP-12, in perpetuating UC inflammation. Moreover, by single-cell RNAseq, they were also able to show that both activated fibroblasts and epithelial cells express *ADM* and that interferon γ is a key upstream regulator of MC gene expression [68], thus defining a new potential therapeutic target. 

## 5. Exploring Intestinal Mast Cells’ Role in Tumor Biology: Colon Cancer under RNAseq Microscope 

MCs’ physiologic function in tumor biology has raised particular interest for decades since these cells potentially influence different aspects of tumorigenesis including angiogenesis, invasiveness, and immunosuppression [69,70,71]. However, the MC contributions in cancer initiation and progression remain controversial [71]. Indeed, several studies have demonstrated both positive and negative correlation of MCs in the development of different types of cancers, including colorectal cancer (CRC) [72,73,74]. 

CRC is the third most common type of malignancy that affects the colon or rectum [75]. Most CRC cases emerge sporadically, while up to 20% of cases present a familial history including familial adenomatous polyposis and Lynch syndrome [76,77]. Moreover, lifestyle as well as chronic inflammation represent independent risk factors for CRC development in patients with IBD [78]. 

The cross-talk between cancer cells and surrounding stromal cells in the tumor microenvironment (TME) and cancer metabolic reprograming also influence the development of CRC [79,80].

The density of tumor-infiltrating cytotoxic and memory T cells, which are associated with a better prognosis, defines the “immunoscore” as an additional parameter to classify CRC [81]. However, the knowledge about innate immune cell infiltration, including MCs, is limited [74,82]. Recent advancements in sequencing approaches have provided crucial opportunities to dissect the heterogeneity and functional role of MCs within the CRC microenvironment and adjacent normal tissue.

By examining the transcriptomic profile among wild-type (WT) mice, MC-deficient mice (Kit^W-sh^), and Kit^W-sh^ mice engrafted with MCs derived from WT mice, Ko and coauthors identified several genes downregulated in the absence of MCs but recovered by MC engraftment [83]. These genes, named “mast cell-dependent genes”, were found to be associated with pathways related to cancer progression including immunosuppression, apoptosis, and angiogenesis. Interestingly, these pathways were enriched in lung, breast, and colon cancer compared to normal tissues, supporting a pro-angiogenic and anti-apoptotic role for MCs in tumor microenvironments. Moreover, genes associated with lymphocyte cytotoxicity were upregulated in the absence of MCs, suggesting that these cells promote immunosuppression [83]. These results support in vitro and in vivo evidence demonstrating a role for tumor-infiltrating MCs in favoring a suppressive microenvironment and/or in promoting tumor growth [69,84,85,86,87]. 

However, by an RNAseq approach, Sakita and coauthors showed that MCs’ role in colon cancer development and progression is multifaceted and context-dependent [86]. Indeed, in a model of spontaneous CRC, MC deficiency promoted tumor development, whereas in colitis-dependent CRC, the absence of MCs reduced tumor burden and increased the frequency of tumor-infiltrating CD8^+^ T cells [86]. Bulk RNAseq analysis of colitis-dependent tumor masses showed that MC deficiency upregulated the cytokine–cytokine receptor pathways, further supporting a role for MCs in suppressing immune responses during tumorigenesis [86]. 

Thus, a characterization of murine CRC-infiltrating MCs and their role in tumor progression is currently unclear. Moreover, whether different MC subsets may play an antitumorigenic or protumorigenic role in different stages of the disease is still unknown.

Using a murine colitis-dependent model of tumorigenesis, we have recently demonstrated that tumor masses are enriched by CTMCs showing an activated phenotype [87]. However, a single-cell RNAseq approach is necessary to better define tumor-infiltrating MC subsets and to compare them in different murine models of CRC. 

Regarding human CRC, several groups have profiled immune and non-immune cells isolated from tumoral lesions [88,89,90,91,92,93,94,95,96,97,98,99,100]. A discrete MC population was identified in the TME based on a unique set of genes including those coding for c-Kit receptor (*KIT*), chymase (*CMA1*), carboxypeptidase 3 (*CPA3*), and tryptases (*TPSAB2* and *TPSB2*) [88,89,90,91,92,93,94,95,96,97,98,99,100]. 

However, there is still inconsistency regarding the real frequency of MCs in transformed and not-transformed tissue and their pro/antitumor activity (Table 1). 

Notably, the different isolation procedures may influence the number and the quality of the cells used to generate single-cell data. Moreover, the methods employed to prepare the library, the use of diverse sequence platforms, and the sequencing depth can lead to different outcomes. Finally, the diverse thresholds used to exclude dead cells and duplets can induce variability in clustering results and data visualization.

Regarding MC numbers, two studies reported a reduced MC frequency in tumor lesions with respect to healthy tissues [94,99]. However, the first study was performed by transcriptomic profiling a very low number of CRC patients [94], and the second result was obtained by a bioinformatic analysis of bulk RNAseq datasets [99].

On the other hand, by employing a single-cell RNAseq approach, two different research groups demonstrated a comparable MC number in both tumors and normal mucosa [89,90], while single-cell analysis of tumor-infiltrating immune cells demonstrated accumulation of MCs in different kinds of cancers, including CRC, compared to nontumoral adjacent tissue [93]. Furthermore, a higher number of MCs was also reported by other groups in advanced CRC stages [92] and in right-sided tumors compared to the left part of the colon [96]. 

A limit of all these data is that they were obtained by the analysis of CRC biopsies, which are a small portion of the tumor mass. Moreover, in most studies, a stratification in different CRC stages was not performed. Of note, the accumulation of MCs was only observed in the late stages [92]. Thus, further research is necessary to clarify these aspects. 

Regarding the role of MCs in human CRC, few data are currently available (Table 1).

Sakita and co-workers found a negative correlation in CRC between the number of activated MCs and infiltrating CD8^+^ T lymphocytes, supporting a protumoral role for MCs [86].

Cheng and coauthors performed a meta-analysis by combined previously published and newly generated sc-RNAseq datasets to compare transcriptomic signatures associated with MCs infiltrating different cancer types [93]. Focusing on CRC patients, they found a down-modulation of *TNFA* transcript and an upregulation of *VEGFA* with respect to adjacent healthy tissue, associating this signature with a decreased patient survival rate. However, the implication of a selective MC subset (mucosal vs connective) was not investigated. 

By an integrated analysis of different CRC datasets, Xie and coauthors revealed the presence of distinct activation MC features in tumor lesions, including high expression of transcripts for specific receptors and mediators as well as transcripts related to the TNFA-NFKB pathway [99]. In addition, they found a positive correlation between the MC activation state and a good CRC prognosis [99], supporting a protective role of MCs during tumor progression. 

These discrepant results may depend on the existence of phenotypical and functional heterogeneity between active MC clusters. Thus, single-cell RNAseq analysis performed after cell sorting could reveal unique MC clusters associated with CRC development and progression. Moreover, patients’ stratification into distinct tumor stages could help to understand whether different MC subsets are involved at the onset of intestinal transformation and in more advanced stages.

Regarding a potential interplay between MCs and other cells in the TME, Wang and coauthors conducted cell–cell communication analysis mapping the expression of ligand–receptor pairs. Their finding highlighted a possible MC interaction with B cells, epithelial cells, and fibroblasts [100]. Notably, MC co-localization with fibroblasts and endothelial cells was also reported in the stromal region of CRC tissue by spatial transcriptomic analysis [99].

Thus, in future studies, the exact localization of MCs within tumor tissue and their interaction with different cell types in CRC could be clarified by integrating single-cell with spatial transcriptomic analysis.

## 6. Conclusions and Future Perspectives

MCs are innate immune cells distributed in all tissues and particularly abundant in the intestine, where they play different roles in homeostasis as well as in inflammatory diseases. Moreover, the increase in MCs in different tumors including colonic tumors has been demonstrated in recent years. MCs are characterized by a vast heterogeneity among tissues, and their phenotypical and functional plasticity allow them to respond to different environmental stimuli. However, whether distinct MC subsets are involved in intestinal diseases and their functions are poorly understood.

The advent of single-cell RNAseq platforms has provided a step forward in the understanding of many biological processes and in the definition of cell functions. Several aspects of MC origin and differentiation into peripheral tissues have been elucidated. 

Even though MCs represent an abundant population in healthy intestines, their number appeared to be increased during inflammation. It could be interesting to clarify whether and how the recruitment of new progenitors contributes to the expansion of MCs during inflammation. Moreover, the role of classical MMC and CTMC subsets in different inflammatory states including allergy to food antigens, parasite infections, or autoinflammatory diseases is still poorly investigated. It is also largely unknown whether MC populations with unique phenotypes and functions arise during inflammation. 

In regard to MCs’ role during colonic transformation, it is still unknown how the tumor microenvironment shapes MC plasticity in terms of phenotype and function and whether unique MC subset(s) differentiate in diverse stages of progression. As discussed above, single-cell RNAseq analysis performed on sorted MCs could help to solve discrepant results but also to discriminate between different MC clusters and subclusters associated with CRC development and progression.

Finally, MCs are located near nerves, and the bidirectional interaction of MCs with the enteric nervous system plays an important role in gastrointestinal inflammation. It could be interesting to investigate whether these interactions are also involved in tumor progression.

Spatial transcriptomic analysis combined with single-cell RNAseq could help to decipher MC cross-talk with the nervous system as well as additional MC interactions in the TME and with the construction of an immune landscape for CRC.

A better characterization of intestinal MCs at various stages of gut inflammation and tumorigenesis would help to define novel potential targets for a therapeutic intervention.

## Figures and Tables

**Table 1:** MC characterization by single-cell RNAseq analysis of human CRC samples.

Sample Type	Cells	Method	MC Frequency	MC Function	Ref.
CRC biopsies and adjacent tissues	Immune cells	10X Genomics Smart-seq2	Comparable frequency	n.d.	Zhang et al., 2020 [90]
CRC biopsies (different stages) and adjacent tissues	Immune cells	Smart-seq2 DNBelab C4	Increased frequency in advanced stages	n.d.	Wang W et al., 2021 [92]
CRC biopsies and adjacent tissues	Immune cells	10X Genomics Analysis of published datasets	Increased frequency in CRC	Protumoral activity	Cheng et al., 2021 [93]
CRC biopsies and liver metastasis	Immune cells	10X Genomics Smart-seq2	Increased in metastasis	Protumoral activity	Liu et al., 2022 [98]
CRC biopsies and adjacent tissues	Immune cells	Analysis of published datasets	n.d.	Protumoral activity	Sakita et al., 2022 [86]
CRC biopsies and adjacent tissues	Immune, epithelial, and stromal cells	10X Genomics	Comparable frequency	n.d.	Lee et al., 2020 [89]
CRC biopsies and adjacent tissues	Immune, epithelial, and stromal cells	10X Genomics	Reduced frequency in CRC	n.d.	Becker et al., 2022 [94]
CRC biopsies and adjacent tissues	Immune, epithelial, and stromal cells	Analysis of published datasets	Reduced frequency in CRC	Antitumoral activity	Xie et al., 2023 [99]
CRC biopsies and adjacent tissues	Immune, epithelial, and stromal cells	Analysis of published datasets	Increased frequency in CRC	Protumoral activity	Wang Q et al., 2023 [100]
CRC biopsies (LCC and RCC) and adjacent tissues	Immune, epithelial, and stromal cells	10X Genomics	Increased frequency in RCC	n.d.	Guo W et al., 2022 [96]
CRC biopsies (LCC and RCC) and adjacent tissues	Immune, epithelial, and stromal cells	Analysis of published datasets	n.d.	Antitumoral activity	Guo JN et al., 2022 [97]

LCC: left-sided colon cancer; RCC: right-sided colon cancer.
